# Measurable Residual Disease in High-Risk Acute Myeloid Leukemia

**DOI:** 10.3390/cancers14051278

**Published:** 2022-03-01

**Authors:** Thomas Cluzeau, Roberto M. Lemoli, James McCloskey, Todd Cooper

**Affiliations:** 1 Service d’hématologie, Université Cote d’Azur, CHU de Nice, 06200 Nice, France; 2 IRCCS Ospedale Policlinico San Martino, 16132 Genoa, Italy; roberto.lemoli@unige.it; 3 Clinic of Hematology, Department of Internal Medicine (DIMI), University of Genoa, 16132 Genoa, Italy; 4 John Theurer Cancer Center, Hackensack University Medical Center, Hackensack, NJ 07601, USA; james.mccloskey@hmhn.org; 5 Division of Hematology/Oncology, Cancer and Blood Disorders Center, Seattle Children’s Hospital, Seattle, WA 98105, USA; todd.cooper@seattlechildrens.org

**Keywords:** acute myeloid leukemia, adult, measurable residual disease, pediatric, prognosis, remission induction

## Abstract

**Simple Summary:**

Assessment of measurable residual disease (MRD) identifies small numbers of acute myeloid leukemia (AML) cells that may remain after initiating treatment. The achievement of MRD negativity (no detectable AML cells remaining) typically predicts better outcomes for patients with AML. Some patients with AML have disease characteristics that put them at a higher risk of treatment failure or relapse; while outcomes for patients with high-risk AML are historically poor with traditional chemotherapy regimens, newer chemotherapy formulations (i.e., CPX-351) and targeted therapies may be more effective in achieving MRD negativity in these patients. Currently, there is no agreement on the best method for determining whether a patient has achieved MRD negativity, and the use of several different methods makes it difficult to compare outcomes across studies. Despite these challenges, regular monitoring of patients for the achievement of MRD negativity will become increasingly important in the routine management of patients with high-risk AML.

**Abstract:**

Mounting evidence suggests measurable residual disease (MRD) assessments are prognostic in acute myeloid leukemia (AML). High-risk AML encompasses a subset of AML with poor response to therapy and prognosis, with features such as therapy-related AML, an antecedent hematologic disorder, extramedullary disease (in adults), and selected mutations and cytogenetic abnormalities. Historically, few patients with high-risk AML achieved deep and durable remission with conventional chemotherapy; however, newer agents might be more effective in achieving MRD-negative remission. CPX-351 (dual-drug liposomal encapsulation of daunorubicin/cytarabine at a synergistic ratio) demonstrated MRD-negativity rates of 36–64% across retrospective studies in adults with newly diagnosed high-risk AML and 84% in pediatric patients with first-relapse AML. Venetoclax (BCL2 inhibitor) demonstrated MRD-negativity rates of 33–53% in combination with hypomethylating agents for high-risk subgroups in studies of older adults with newly diagnosed AML who were ineligible for intensive therapy and 65% in combination with chemotherapy in pediatric patients with relapsed/refractory AML. However, there is no consensus on optimal MRD methodology in AML, and the use of different techniques, sample sources, sensitivity thresholds, and the timing of assessments limit comparisons across studies. Robust MRD analyses are needed in future clinical studies, and MRD monitoring should become a routine aspect of AML management.

## 1. Introduction

Morphologic assessments are often used to determine response to treatment in acute myeloid leukemia (AML) [1]. However, many patients who achieve morphologic complete remission (CR) eventually relapse, highlighting the need for more sensitive measurements of remission [1]. Additionally, morphologic assessments in pediatric patients with AML are often inconsistent and difficult to assess [2], further emphasizing the importance of more accurate remission measurements. Measurable residual disease (MRD) assessment allows clinicians to detect disease below the 5% threshold previously used in the morphology-based determination of disease status [3]. MRD can be used to determine the depth of remission, predict a patient’s response to treatment, and monitor a patient for potential relapse [4]. While MRD is well standardized in a variety of hematologic malignancies, including acute lymphocytic leukemia [5] and chronic leukemias (chronic lymphocytic leukemia and chronic myeloid leukemia) [6], and is routinely used in pediatric AML, its utility in the adult AML field is emerging. In 2017, the European LeukemiaNet recommended that MRD negativity should be considered a response criterion in AML [7]. However, despite mounting evidence that MRD assessments are prognostic in AML, they are not standardly used in clinical practice for adults, and many clinical trial protocols do not require MRD assessment.

High-risk AML encompasses a subset of AML with poor response to therapy and prognosis. High-risk features are defined by cytogenetic and molecular features, as well as response to therapy. Recent advances in genomic testing have identified that pediatric and adult AML populations are biologically distinct, with adults having a higher prevalence of mutations such as *IDH1/2* and *DNMT3A* and pediatric patients having a higher prevalence of cryptic fusions and translocations [8]. However, there are subsets of patients in the adult and pediatric AML populations that have high-risk features, and older patients are more commonly affected by high-risk disease. Features of high-risk AML include therapy-related AML, which is developed as a late complication of chemotherapy or ionizing radiation [9]; AML with an antecedent hematologic disorder, such as myelodysplastic syndrome or myeloproliferative neoplasms; and the presence of extramedullary disease (in adults only) [10]. In addition, selected mutations and cytogenetic features define high-risk disease at diagnosis [7,10]. Many patients with high-risk AML benefit from allogeneic hematopoietic cell transplantation (HCT), and several published papers have demonstrated better outcomes for patients transplanted after achieving MRD-negative CR [11,12,13,14,15,16,17,18,19,20]. Additionally, novel therapies may induce MRD-negative CR in a high proportion of patients. Thus, MRD responses have important implications for transplantation selection and optimal bridging to HCT.

In this article, we review the importance and the current landscape of MRD assessment in patients with high-risk AML, focusing on the adult subsets of therapy-related AML, AML with myelodysplasia-related changes (including an antecedent hematologic disorder), and adverse-risk AML per the European LeukemiaNet and National Comprehensive Cancer Network classifications, and on pediatric patients regarding the protocols for high-risk disease. Other high-risk AML subtypes are not discussed due to the lack of MRD data.

## 2. Methods of MRD Detection

Various techniques are currently utilized to assess MRD in AML, and these are summarized in Table 1. Fluorescence in situ hybridization (FISH)-based methods assess chromosomal abnormalities, while multiparameter flow cytometry (MFC) is used to detect aberrantly expressed antigens that differentiate leukemic cells from normal bone marrow cells [1,21]. There are two different approaches used in MFC: the leukemia-associated immunophenotypes (LAIP) approach identifies a unique immunophenotype of leukemic blasts at diagnosis that can be assessed over time; in contrast, the different-from-normal (DfN) approach identifies aberrant differentiation/maturation profiles, which allows for the monitoring of patients during follow up [3,22]. To accommodate potential immunophenotypic shifts due to clonal evolution, the European LeukemiaNet recommends a combination of both approaches be used [4].

Molecular approaches are also frequently used to assess MRD: polymerase chain reaction (PCR)-based methods (including digital PCR) monitor changes in particular genes where real-time quantitative PCR (qPCR) platforms are developed (e.g., the detection of *NPM1* mutation), whereas next-generation sequencing (NGS) examines a panel of genes of interest at once [1]. NGS MRD can be detected at a 0.001% to 0.0001% threshold in the research setting, although this is not currently achievable in clinical practice (typically only 1 to 2%) [1]. The development of NGS panels more specifically designed for MRD assessment may improve its clinical utility. The clinical significance of NGS MRD is further challenged by the difficulty in differentiating between pre-leukemic clonal hematopoiesis and malignant clones [24]. Mutations in DTA (i.e., mutations in clonal hematopoiesis-associated genes *DNMT3A*, *TET2*, and *ASXL1*), CHIP (i.e., clonal hematopoiesis of indeterminate potential), or in pre-leukemic diseases such as myelodysplastic syndrome may not reflect residual AML [24,25]. Therefore, disregarding these mutations from the sequencing panels can enhance the predictive power of MRD assessments [24]; however, recommendations regarding specific mutations are still uncertain. One highly sensitive NGS technology that may address these limitations is high throughput single-cell sequencing (SCS), which can evaluate the clonal dynamics of AML from diagnosis to remission to relapse. The preliminary clinical validation of the utility of SCS in AML was provided by Ediriwickrema et al. [24]. In this study, SCS of AML samples at diagnosis, remission, and relapse allowed for quantification of co-occurring mutation variants, differentiation of pre-leukemic clonal hematopoiesis from relapse-causing clones, and identification of clinically relevant MRD, suggesting the future applicability of SCS in clinical practice. As technology continues to evolve, NGS may become a preferred method of MRD assessment due to its ability to simultaneously detect multiple leukemia-specific aberrations.

## 3. Prognostic Value of MRD in AML

Several new therapies were approved for the treatment of AML in recent years, many of which may induce deeper responses, sparking an interest in assessing MRD to improve the management of patients with AML. Numerous studies have since demonstrated the predictive nature of MRD assessment in patients with AML both in adult and in pediatric patient populations; these studies are summarized in Table 2. For example, in a meta-analysis of 81 studies reporting on 11,151 adults with AML, patients with MRD negativity had improved rates of overall survival (OS; 68% vs. 34%) and disease-free survival (64% vs. 25%) at 5 years versus patients with MRD positivity [26]. However, it should be noted that few data are available in patients with high-risk AML. Additionally, results from a retrospective study supported serial NGS assessments as a clinically robust tool for the evaluation of prognosis in patients with secondary AML [27]. In this study, patients achieving NGS negativity had significantly improved median OS versus those with mutation persistence (not reached vs. 18.5 months; *p* = 0.002).

Several studies also demonstrated that adult and pediatric patients with AML who retain MRD positivity after induction treatment have worse outcomes following HCT compared to patients with MRD negativity (Table 2). For example, in a meta-analysis of 19 studies reporting on 1431 patients, MRD positivity before HCT was associated with decreased leukemia-free survival (hazard ratio [HR] = 2.76), OS (HR = 2.36), and cumulative incidence of relapse (CIR; HR = 3.65) [16]. Despite these findings, it should be noted that, in current clinical practice, patients with high-risk AML are considered candidates for HCT regardless of MRD assessment. However, patients with MRD positivity may benefit from post-transplantation strategies, such as maintenance therapy or preemptive immune therapy [11]. MRD analysis may also influence the selection of the conditioning regimen for HCT. For example, Hourigan et al. demonstrated that reduced-intensity conditioning resulted in worse outcomes when compared to full conditioning in patients with MRD positivity [35].

## 4. Limitations and Challenges in MRD Assessment in AML

Since MRD assessments are not routinely used in AML, there are inconsistencies in procedure between studies, complicating inter-study comparisons. The sample source often differs between studies; while some studies perform MRD assessment on peripheral blood, others use bone marrow samples [36]. Although analyses have shown bone marrow to have higher MRD levels and higher sensitivity, peripheral blood sample collection is more convenient for the patient [36]. The European LeukemiaNet working group recommends the use of first pull bone marrow aspirate for MFC MRD assessments, but either bone marrow or peripheral blood samples for molecular MRD assessments [4].

There is a large degree of variability regarding the timing of MRD assessments used in clinical studies [36]. At this time, there is limited consensus on the optimal timing of MRD assessments across methods and targets. The European LeukemiaNet working group recommends assessments at diagnosis, after two cycles of induction/consolidation, prior to HCT, and at the end of treatment [4], although the optimal timing may vary depending on the induction/consolidation regimen, patient population, and type of MRD assessment. For example, in patients treated with fludarabine, cytarabine, and idarubicin induction, MFC MRD assessments performed after the first induction were most predictive of clinical outcomes [37]. Another study in pediatric patients with high-risk AML treated with cytarabine, daunorubicin, and etoposide also found that MFC MRD analysis at the end of the first induction was most predictive of outcomes [32]. However, NGS MRD assessments performed at first consolidation were predictive of 5-year clinical outcomes in a study in adults with AML who were treated with standard induction chemotherapy [38].

There is also no standardized, predictive threshold for MRD negativity, with different thresholds used for different MRD methods [36]. The European LeukemiaNet working group defines MFC MRD test positivity as ≥0.1% of CD45-expressing cells with the target immunophenotype and qPCR MRD test positivity as a cycling threshold of <40 in at least two of three replicates [4]. The optimal NGS MRD threshold is yet to be defined. Furthermore, methodology for AML assessments often varies between sites and studies; there are not many centralized studies that incorporate consistent methodology, and some methods are currently being explored/used by specific groups without broad clinical use. More studies are thus needed to optimize and standardize MRD assessments in AML.

## 5. Promising Strategies to Achieve MRD Negativity in High-Risk AML

Although intensive chemotherapy followed by HCT is generally recognized as offering the best chance for long-term remission, historically, few patients with high-risk AML have achieved deep and durable remission with conventional chemotherapy regimens. Some novel chemotherapy formulations and regimens may be more effective in achieving MRD-negative remission in this population. The combination of targeted therapies, such as FLT3 inhibitors, with intensive chemotherapy is also a promising strategy to improve the depth of remission and outcomes in patients who have targetable mutations. Refinements in HCT conditioning regimens may also help to deepen and/or prolong remissions prior to and after transplantation. Unfortunately, limited MRD data are published for patients with high-risk subtypes of AML (Table 3).

### 5.1. CPX-351 in High-Risk AML

CPX-351 is a dual-drug liposomal encapsulation of daunorubicin and cytarabine in a 1:5 molar drug ratio. Based on results from a phase 3 study of CPX-351 versus conventional 7 + 3 chemotherapy in older adults (aged 60 to 75 years) with newly diagnosed secondary AML, CPX-351 was approved for the treatment of newly diagnosed therapy-related AML and AML with myelodysplasia-related changes (AML-MRC). The phase 3 study protocol, unfortunately, did not include MRD assessment; however, a few non-randomized studies with relatively small sample sizes have assessed MRD status after CPX-351 treatment. In an Italian compassionate use program of CPX-351 in older adults with newly diagnosed therapy-related AML or AML-MRC [39], MRD negativity was observed in 38% and 54% of patients assessed with MFC (n = 40) and *WT1* qPCR (n = 38) methods, respectively. Although not statistically significant, a trend for MRD negativity and improved 12-month CIR (11% vs. 37% for MRD-negative and MRD-positive patients, respectively; *p* = 0.151) was observed; however, 12-month OS was 71% versus 84% for MRD-negative and MRD-positive patients, respectively (*p* = 0.414) [39]. In a French retrospective, multicenter analysis in patients with newly diagnosed therapy-related AML or AML-MRC [40], 28 patients who achieved CR or CR with incomplete hematologic recovery (CRi) underwent MRD analysis (by NGS, MFC, or qPCR). Among those 28 responding patients, 16 (57%) achieved MRD negativity (defined as < 10^−3^), including 8/14 (57%) patients who had available MRD data and proceeded to HCT; so far, OS among transplanted patients was not significantly different between patients with versus without MRD negativity [40]. In a German retrospective, multicenter analysis in patients with newly diagnosed therapy-related AML or AML-MRC [41], a total of 36 patients who achieved CR or CRi underwent MRD analysis (by MFC), and 23/36 (64%) achieved MRD negativity (defined as < 10^−3^); further, all 23 patients proceeded to HCT and continued to exhibit MRD negativity at the time of HCT [41]. The achievement of MRD negativity was associated with longer OS overall (*p* = 0.01) and among patients who proceeded to HCT (*p* = 0.02), but not in a multivariable analysis for OS [41]. Although these real-world studies found no consistent difference in OS between patients with versus without MRD negativity, it should be noted that their median follow-up times were relatively short (8.6 to 11 months). In a single-institution retrospective analysis of patients with therapy-related AML or AML-MRC, MRD negativity (based on MFC; n = 34) was achieved by 12/23 (52%) responders [43]. Finally, in a multicenter retrospective analysis of patients with therapy-related AML or AML-MRC, MRD negativity (based on MFC; n = 37) was achieved at a higher rate in patients with wild-type versus mutated *TP53* (36% vs. 8%) [42].

In the Children’s Oncology Group (COG) AAML1421 study of pediatric patients with AML in first relapse who were treated with CPX-351 followed by FLAG chemotherapy (fludarabine, cytarabine, and granulocyte colony-stimulating factor; N = 38, including 24 [63%] who had relapsed within 1 year), 16 patients achieved CR or CR with incomplete platelet recovery (CRp) after an initial cycle of CPX-351 treatment, 12 (75%) of whom achieved MRD negativity (based on MFC) [44]. Of the 25 patients who achieved CR or CRp as their best response after receiving CPX-351 and the second cycle of FLAG treatment, 21/25 (84%) achieved MRD negativity; additionally, 24/30 (80%) patients with CR, CRp, or CRi achieved MRD negativity. Notably, MRD determination was not centralized and instead carried out according to individual, institutional practices.

In the phase 3 study of CPX-351 versus conventional 7 + 3 chemotherapy, CPX-351 improved median OS among patients who achieved CR or CRi, as well as the likelihood of proceeding to HCT and median OS landmarked from the date of HCT [51,52], suggesting the potential for achievement of deeper responses with CPX-351. However, MRD is not yet assessed in the context of a large, randomized study of CPX-351 treatment, although real-world data on MRD assessment after CPX-351 treatment are encouraging. Patients with newly diagnosed AML with intermediate- or adverse-risk genetics are currently being recruited for a phase 3 study in Germany comparing CPX-351 to conventional intensive chemotherapy (ClinicalTrials.gov Identifier: NCT03897127). The primary endpoint of the study is event-free survival, and the secondary endpoints are OS, relapse-free survival, CIR, the cumulative incidence of death, rate of objective response (including MRD-negative CR), and adverse events. Further, a large, randomized study of CPX-351 (COG AAML1831; NCT04293562) in patients with newly diagnosed AML is currently recruiting patients. This study will incorporate central MRD determination at the end of induction one and induction two using the DfN methodology.

### 5.2. Venetoclax Combinations in High-Risk AML

Venetoclax is an oral BCL2 inhibitor approved in combination with a hypomethylating agent or low-dose cytarabine (the United States only) for the treatment of newly diagnosed AML in adults who are considered ineligible for intensive induction chemotherapy. Although treatment with intensive chemotherapy followed by HCT provides the greatest likelihood of achieving long-term remission, some patients do achieve MRD negativity with less-intensive therapies. The phase 3 VIALE-A trial evaluated venetoclax combined with azacitidine (n = 286) versus azacitidine alone (n = 145) in adult patients with newly diagnosed AML who were considered ineligible for intensive chemotherapy; 25% of patients had secondary AML, 33% had AML-MRC, and 37% had poor-risk cytogenetics [46]. In this study, MRD negativity (<10^−3^ based on MFC) occurred in 67/164 (41%) of evaluable patients who achieved CR or CRi, including 18/44 (41%) patients with secondary AML and 16/48 (33%) patients with poor-risk cytogenetics [47]. Those with MRD negativity had improved 12-month duration of response (81% vs. 47%), OS (94% vs. 68%), and event-free survival (83% vs. 45%) versus those with MRD ≥ 10^−3^, and these results were confirmed among patients with secondary AML or poor-risk cytogenetics [47]. In a study of 118 older adults with newly diagnosed AML (57% of whom had European LeukemiaNet adverse-risk disease) who were considered ineligible for intensive chemotherapy, venetoclax plus 10-day decitabine induced MRD negativity based on MFC in 54% of responding patients [45]. Rates of MRD negativity in patients with secondary AML (prior treatment for antecedent disorder: 53%; no prior treatment: 42%), therapy-related AML (38%), and adverse-risk cytogenetics (33%) were lower than those with de novo AML (62%) or intermediate-risk cytogenetics (67%). Patients who achieved MRD negativity within 2 months had improved median relapse-free survival (not reached vs. 5.2 months), event-free survival (not reached vs. 5.8 months), and OS (25.1 vs. 7.1 months) compared to patients who remained MRD positive. In an interim analysis of a separate ongoing, phase 2 study of eight patients aged < 60 years with adverse-risk AML, five of six responders to venetoclax and azacitidine achieved MRD negativity by MFC, and one of six responders achieved MRD negativity by droplet digital qPCR [48].

In a phase 1 study in pediatric patients with relapsed/refractory AML who were treated with venetoclax in combination with cytarabine with or without idarubicin, 20 patients achieved CR or CRi, 13 of whom achieved MRD negativity (based on flow cytometry by central assessment) [49].

Although MRD data are available for venetoclax combination regimens, including data from a randomized study, these data are for a mixed population that includes a large proportion of patients who were not identified as having high-risk subtypes of AML. Further, these patients were also identified as not being suitable for intensive chemotherapy, where the relevance of MRD as a surrogate endpoint is still to be determined. Similar to the case with CPX-351, robust MRD data are needed for venetoclax regimens to help guide treatment decisions for this population.

### 5.3. Other Regimens in High-Risk AML

In a study of 244 patients with high-risk AML or myelodysplastic syndrome undergoing their first HCT, sequential transplantation regimen FLAMSA-Bu (fludarabine/amsacrine/cytarabine-busulfan) was compared with a fludarabine-based reduced-intensity conditioning regimen [50]. MRD positivity (based on MFC) was detected in 38% of patients receiving FLAMSA-Bu and 48% receiving fludarabine-based reduced-intensity conditioning. In all randomized patients, pre-HCT MRD positivity was associated with an increased risk of 2-year CIR (41% vs. 20%) and a reduction in 2-year OS (51% vs. 70%) [50]. In another study in patients with AML who underwent allogeneic HCT (27% with secondary AML, 47% with adverse-risk AML), the monitoring of DTA mutations as potential NGS MRD markers was not prognostic for outcomes, although non-DTA mutations were prognostic for CIR, relapse-free survival, and OS after HCT [18]. However, specific treatment regimens received prior to HCT were not described.

### 5.4. Maintenance Therapy in High-Risk AML

The objective of maintenance therapy is to maintain MRD negativity, reduce the risk of relapse, and prolong survival. The phase 3 QUAZAR AML-001 trial found that CC-486 (oral azacitidine; n = 238) maintenance therapy significantly prolonged OS and relapse-free survival versus placebo (n = 234) irrespective of MRD status in patients with AML who were in the first remission after intensive chemotherapy [53]. While maintenance therapy may benefit patients with high-risk AML, it should be noted that only a small proportion of patients in this study had high-risk disease features.

## 6. Future Perspectives and Conclusions

Although recent evidence suggests MRD assessments can be predictive of clinical outcomes in patients with favorable- or intermediate-risk AML, the results for patients with high-risk disease remain controversial and warrant further investigation. Additional studies are also needed to optimize and standardize the methodology and timing of MRD assessment in patients with AML, particularly those with high-risk disease. Further, the relevance of MRD as a prognostic indicator may be different in patients with high-risk AML who receive less-intensive therapy instead of intensive chemotherapy as induction due to fitness status. While MRD data were inconsistently collected in past clinical trials in AML, future clinical trials should include robust MRD analyses, as it is likely that CR with MRD negativity will become a key efficacy endpoint in the new drug era. MRD monitoring should also become a more routine aspect of AML management outside of clinical trials, as it can provide important information on patient outcomes and help to inform treatment decisions, such as the selection of HCT conditioning regimen and the need for post-HCT strategies in patients with MRD-positive disease. Achieving MRD negativity should be the main goal of novel therapies, and combination regimens may be more effective at inducing MRD negativity.

## Figures and Tables

**Table 1:** Methods of MRD detection [1,4,21].

Method	Target	Markers	Sensitivity *	Strengths	Weaknesses
**FISH**	Chromosomal aberrations	N/A	1 to 2%	Widely available Detection of numeric cytogenetic abnormalities	Insensitive
**MFC ^†^**	Leukemia-associated aberrant immunophenotypes	CD2, CD4, CD7, CD13, CD15, CD19, CD33, CD34, CD38, CD45, CD56, CD117, CD123, HLA-DR LSCs ^‡^ are CD34+/CD38- cells combined with an aberrant marker not present on HSCs (e.g., CD45RA, CLL-1, CD123) [4]	1 in 1000 (0.1%) to 1 in 10,000 (0.01%)	Wide applicability (>90%) Relatively quick High specificity and sensitivity Leukemia stem cell phenotype	Challenging Experience-dependent Dependent on antibody panel Limited standardization Phenotype not always stable
**PCR**	Fusion transcripts Gene mutations Overexpressed genes	*CBFB-MYH11, IDH1/IDH2, NPM1, RUNX1/RUNX1T1,KMT2A* (various)*, WT1, PML-RARα, BCR-ABL, DEK-NUP214*	1 in 10,000 (0.01%) to 1 in 1,000,000 (0.0001%)	Wide applicability High sensitivity Well standardized	Multiple days Expensive Applicable to only ~50% of cases
**NGS**	Gene mutations	*NPM1*, *FLT3*-ITD, *IDH1/IDH2*; some panels can examine hundreds of genes of interest [23]	1 in 100,000 (0.001%) to 1 in 1,000,000 (0.0001%) ^§^	Relatively easy to perform Sensitive	Limited standardization CHIP-mutated genes Persistent mutants in CR

CHIP, clonal hematopoiesis of indeterminate potential; CR, complete remission; FISH, fluorescence in situ hybridization; ITD, internal tandem duplication; MFC, multiparameter flow cytometry; MRD, measurable residual disease; N/A, not applicable; NGS, next-generation sequencing; LSC, leukemia stem cell; HSC, hematopoietic stem cell; PCR, polymerase chain reaction. * Thresholds considered to be routinely achievable in clinical practice. ^†^ The leukemia-associated aberrant immunophenotype approach defines leukemia-associated aberrant immunophenotypes at diagnosis and tracks these over time, whereas the different-from-normal approach is based on the identification of aberrant differentiation/maturation profiles at follow-up. ^‡^ The European LeukemiaNet recommends further validation of LSCs in prospective clinical trials, as measurements of LSCs may have a prognostic value [4]. ^§^ Achievable only in the research setting.

**Table 2:** Prognostic Value of MRD in AML.

Study	Regimen	Population	MRD Method	Results *		
				MRD Negative	MRD Positive	*p*-Value
**Prognostic Value in Adults with AML**						
Short 2020 [26]	Review of 81 publications	N = 11,151	MFC, qPCR, NGS, or cytogenetics/FISH in BM or peripheral blood at induction or during/after consolidation	5-y DFS: 64% 5-y OS: 68%	5-y DFS: 25% 5-y OS: 34%	Not reported
Salek 2020 [28]	Intensive chemotherapy	*WT1*-mutated intermediate- or high-risk AML Median age: 56 y for *WT1*-intermediate AML and 51 y for *WT1*-high AML N = 106	qPCR of *WT1* in peripheral blood after two cycles of treatment	3-y OS: 66% 3-y EFS: 45%	3-y OS: 41% 3-y EFS: 22%	3-y OS: *p* = 0.01 3-y EFS: *p* = 0.01
Lambert 2021 [29] ALFA-0702 trial	Daunorubicin plus cytarabine induction with G-CSF; potential salvage with idarubicin and high-dose cytarabine	de novo AML N = 447	qPCR of *WT1* in BM or peripheral blood	4-y CIR: 29% 4-y OS: 71% 4-y RFS: 60%	4-y CIR: 61% 4-y OS: 44% 4-y RFS: 26%	4-y CIR: *p* < 0.0001 4-y OS: *p* = 0.0005 4-y RFS: *p* < 0.0001
**Prognostic value in pediatric patients with AML**						
Langebrake 2006 [30]	Intensive chemotherapy	de novo AML Pediatric patients N = 150	LAIP MFC in BM at BM puncture 1 (median of 15 days from the start of therapy) or BM puncture 2 (median of 29 days from the start of therapy)	3-y EFS after BM puncture 1: 71% 3-y EFS after BM puncture 2: 70% High-risk patients: 3-y EFS after BM puncture 1: 60% 3-y EFS after BM puncture 2: 58%	3-y EFS after BM puncture 1: 48% 3-y EFS after BM puncture 2: 50% High-risk patients: 3-y EFS after BM puncture 1: 43% 3-y EFS after BM puncture 2: 44%	3-y EFS after BM puncture 1: *p* = 0.029 3-y EFS after BM puncture 2: *p* = 0.033 High-risk patients: 3-y EFS after BM puncture 1: *p* = 0.16 3-y EFS after BM puncture 2: *p* = 0.22
Loken 2012 [31] AAML03PI trial	Two courses ofcytarabine, daunorubicin, and etoposide, plus gemtuzumabozogamicin in the first course; additional three courses of intensive chemotherapy	Newly diagnosed de novo AML Patients < 21 y of age N = 249	DfN MFC in BM or peripheral blood at the end of induction 1	3-y relapse risk: 29% 3-y RFS: 65% High-risk patients: 3-y RFS: 45%	3-year relapse risk: 60% 3-year RFS: 30% High-risk patients: 3-y RFS: 0%	3-y relapse risk: *p* < 0.001 3-y RFS: *p* < 0.001 High-risk patients: 3-y RFS: *p* = 0.047
Rubnitz 2010 [32] AML02 trial	High-dose or low-dose cytarabine plus daunorubicin and etoposide	de novo AML, therapy-related AML, MDS-related AML, or mixed-lineage leukemia Median age: 9 y N = 230	LAIP MFC in BM on Day 22 of the first induction	3-y CIR: 17% 3-yr EFS: 74% High-risk patients: 3-y CIR: 21%	3-year CIR: 39% 3-year EFS: 43% High-risk patients: 3-y CIR: 45%	3-y CIR: *p* < 0.0001 3-y EFS: *p* < 0.0001 High-risk patients: 3-y CIR: *p* = 0.01
Sievers 1996 [33]	Intensive chemotherapy	Newly diagnosed AML Median age: 8 y N = 39	MFC in BM during CR1	Relapse in 9 of 11 (82%) patients without HCT Median time to relapse: 413 d	Relapse in 13 of 14 (93%) patients without HCT Median time to relapse: 153 d	Relapse risk: *p* = 0.02 for patients with ≤0.1% vs. >0.1% abnormal cells
Sievers 2003 [34] CCG-2941 and CCG-2961 trials	Intensive chemotherapy	Newly diagnosed AML and MDS Pediatric patients N = 252	MFC in BM after induction	MRD positivity was associated with a worsened risk of relapse and death: Relative risk of relapse: 4.8 (*p* < 0.0001) Relative risk of death: 3.1 (*p* < 0.0001)		
**Post-HCT prognostic value in adults with AML**						
Araki 2016 [12]	Myeloablative allogeneic HCT	Median age at HCT: 50 y N = 359	MFC in BM aspirates pre-HCT	3-y CIR: 22% 3-y OS: 73% 3-y PFS: 67% 3-y NRM: 11%	3-y CIR: 67% 3-y OS: 26% 3-y PFS: 12% 3-y NRM: 21%	Not reported
Veltri 2019 [11]	HCT with myeloablative or reduced-intensity conditioning	High-risk AML Median age: 68 y N = 185	MFC in BM pre-HCT	2-y CIR: 18% 2-y OS: 69% 5-y OS: 67%	2-y CIR: 56% 2-y OS: 21% 5-y OS: 8%	2-y CIR: *p* < 0.0001 2-y OS: *p* < 0.0001 5-y OS: *p* < 0.0001
Walter 2011 [13]	Myeloablative HCT	Median age: 45 y N = 99	MFC in BM aspirates pre-HCT	2-y OS: 77% 2-y DFS: 75% 2-y relapse: 18%	2-y OS: 30% 2-y DFS: 9% 2-y relapse: 65%	Not reported
Walter 2013 [14]	Myeloablative HCT in CR1 or CR2	Median age at HCT: 43 y N = 253	MFC in BM aspirates pre-HCT	3-y OS in CR1: 73% 3-y OS in CR2: 73% 3-y relapse risk in CR1: 21% 3-y relapse risk in CR2: 19%	3-y OS in CR1: 32% 3-y OS in CR2: 44% 3-y relapse risk in CR1: 59% 3-y relapse risk in CR2: 68%	Not reported
Walter 2015 [15]	Myeloablative or non-myeloablative HCT	Age range of study: 18–75 y N = 241	MFC in BM aspirates pre-HCT	3-y CIR for myeloablative: 22% 3-y CIR for non-myeloablative: 28% 3-y OS for myeloablative: 76% 3-y OS for non-myeloablative: 48% 3-y DFS for myeloablative: 71% 3-y DFS for non-myeloablative: 42%	3-y CIR for myeloablative: 63% 3-y CIR for non-myeloablative: 57% 3-y OS for myeloablative: 25% 3-y OS for non-myeloablative: 41% 3-y DFS for myeloablative: 13% 3-y DFS for non-myeloablative: 33%	Not reported
Hourigan 2020 [35]	HCT	Age range of study: 22–66 y N = 190	NGS in blood pre-HCT	3-y OS for myeloablative: 56% 3-y OS for RIC: 63%	3-y OS for myeloablative: 61% 3-y OS for RIC: 43% 1-y CIR for myeloablative: 14% 1-y CIR for RIC: 58%	Not reported
Buckley 2017 [16]	Review of 19 publications	N = 1431	MFC, PCR, or cytogenetics/FISH in BM or peripheral blood	MRD positivity was associated with worsened LFS, OS, and CIR: LFS: HR = 2.76 (1.90–4.00) OS: HR = 2.36 (1.73–3.22) CIR: HR = 3.65 (2.53–5.27)		
Heuser 2021 [18]	HCT	Non–DTA-mutated AML Median age: 53 y N = 131	NGS in BM or peripheral blood post-HCT	In a multivariate analysis, MRD positivity adversely predicted CIR, RFS, and OS: CIR: HR = 3.27; *p* = 0.002 RFS: HR = 3.57; *p* < 0.001 OS: HR = 2.18; *p* = 0.028		
**Post-HCT prognostic value in pediatric patients with AML**						
Horan 2013 [19] AAML0531 and AAML03PI trials	HCT in CR1	de novo AML Pediatric patients N = 108	MFC in BM in CR1 pre-HCT	3-y OS: 76% 3-y CIR: 30%	3-y OS: 47% 3-y CIR: 50%	3-y OS: *p* = 0.023 3-y CIR: *p* = 0.037
Jacobsohn 2018 [20]	HCT	Patients < 21 y of age N = 150	DfN MFC in BM pre-HCT	2-y relapse risk: 32% 2-y DFS: 55% 2-y OS: 63%	2-y relapse risk: 70% 2-y DFS: 10% 2-y OS: 20%	2-y relapse risk: *p* = 0.01 2-y DFS: *p* < 0.001 2-y OS: *p* = 0.001

AML, acute myeloid leukemia; BM, bone marrow; CIR, cumulative incidence of relapse; CR1, first complete remission; CR2, second complete remission; DfN, different-from-normal; DFS, disease-free survival; DTA, clonal hematopoiesis–associated genes (DNMT3A, TET2, ASXL1); EFS, event-free survival; FISH, fluorescence in situ hybridization; G-CSF, granulocyte colony-stimulating factor; HCT, hematopoietic cell transplantation; HR, hazard ratio; LAIP, leukemia-associated immunophenotypes; LFS, leukemia-free survival; MDS, myelodysplastic syndrome; MFC, multiparameter flow cytometry; MRD, measurable residual disease; NGS, next-generation sequencing; NRM, non-relapse mortality; OS, overall survival; PCR, polymerase chain reaction; PFS, progression-free survival; qPCR, real-time quantitative polymerase chain reaction; RFS, relapse-free survival; RIC, reduced-intensity conditioning; RT-qPCR, reverse-transcriptase quantitative polymerase chain reaction. * Available data related to MRD in high-risk patients have been included, if applicable.

**Table 3:** Studies including MRD assessments in high-risk AML.

Regimen	Study Design	Population	MRD Assessment	MRD Results *	
				MRD Negativity	MRD Negative Versus MRD Positive
CPX-351 [39]	Italian compassionate use program	Adults with newly diagnosed therapy-related AML or AML-MRC	Assessed by MFC or *WT1* non-centrally by individual clinical practices MRD negativity threshold not reported	MRD negativity by MFC: 38% MRD negativity by *WT1*: 54%	12-mo CIR: 11% vs. 37% (*p* = 0.15) 12-mo OS: 71% vs. 84% (*p* = 0.41)
CPX-351 [40]	Retrospective analysis	Adults with newly diagnosed therapy-related AML or AML-MRC	Assessed by NGS, MFC, or qPCR non-centrally by individual clinical practices MRD negativity threshold: <10^−3^	MRD negativity: 57%	Among patients who proceeded to HCT, OS was not significantly different between patients with vs. without MRD negativity
CPX-351 [41]	Retrospective analysis	Adults with newly diagnosed therapy-related AML or AML-MRC	Assessed by MFC MRD negativity threshold: <10^−3^	MRD negativity: 64%	OS longer in patients with MRD negativity in univariable analysis but not multivariable analysis Among patients who proceeded to HCT, OS longer in patients with MRD negativity in univariable analysis
CPX-351 [42]	Retrospective analysis	Adults with therapy-related AML or AML-MRC	Assessed by MFC non-centrally by individual clinical practices Any level of residual disease was considered MRD positive	MRD negativity in patients with wild-type vs. mutated *TP53*: 36% vs. 8%	Not reported
CPX-351 [43]	Retrospective analysis	Adults with therapy-related AML or AML-MRC	Assessment by MFC (single-center study) MRD negativity threshold: <0.01%	MRD negativity: 52%	Not reported
CPX-351 followed by FLAG [44]	Phase 1/2 study	Pediatric patients with first relapse AML	Assessed by MFC non-centrally by individual clinical practices MRD negativity threshold: not reported	MRD negativity after treatment with CPX-351 followed by FLAG: 84%	Not reported
Venetoclax plus decitabine [45]	Phase 2 study	Older adults with newly diagnosed AML who were considered ineligible for intensive chemotherapy (57% with adverse-risk AML per ELN criteria)	Assessment by MFC (single-center study) MRD negativity threshold: <0.1%	MRD negativity in patients with secondary AML with prior treatment for antecedent disorder: 53% MRD negativity in patients with secondary AML with no prior treatment: 42% MRD negativity in patients with therapy-related AML: 38% MRD negativity in patients with adverse-risk cytogenetics: 33%	Not reported
Venetoclax plus azacitidine [46,47]	Phase 3 study	Adults with Older adults with newly diagnosed AML who were considered ineligible for intensive chemotherapy (25% with secondary AML; 37% with poor-risk cytogenetics)	Assessment by MFC MRD negativity threshold: <10^−3^	MRD negativity in patients with secondary AML: 41% MRD negativity in patients with poor-risk cytogenetics: 33%	Secondary AML ^†^: DOR: HR = 0.40 (0.15, 1.07) OS: HR = 0.35 (0.13, 0.98) EFS: HR = 0.40 (0.17, 0.93) Poor-risk cytogenetics ^†^: DOR: HR = 0.36 (0.15, 0.86) OS: HR = 0.25 (0.09, 0.67) EFS: HR = 0.31 (0.14, 0.70)
Venetoclax plus azacitidine [48]	Phase 2 study	Adults aged < 60 years with adverse-risk AML per ELN criteria	Assessment by MFC or droplet digital qPCR (single-center study) MRD negativity threshold: not reported	MRD negativity by MFC: 5/6 (83%) MRD negativity by droplet digital qPCR: 1/6 (17%)	Not reported
Venetoclax plus cytarabine with or without idarubicin [49]	Phase 1 study	Pediatric patients with relapsed/ refractory AML	Central assessment by flow cytometry MRD negativity threshold: <0.1%	MRD negativity: 65%	Not reported
FLAMSA-Bu (fludarabine/amsacrine/cytarabine-busulfan) vs. fludarabine-based RIC [50]	Phase 2 study	Adults with high-risk AML or MDS undergoing the first HCT	Central assessment by MFC MRD negativity threshold: <0.02–0.05%	MRD positivity vs. fludarabine-based RIC: 38% vs. 48%	2-y CIR: 20% vs. 41% (*p* = 0.01) 2-y OS: 70% vs. 51% (*p* = 0.05)

AML, acute myeloid leukemia; AML-MRC, acute myeloid leukemia with myelodysplasia-related changes; CIR, cumulative incidence of relapse; DOR, duration of response; EFS, event-free survival; ELN, European LeukemiaNet; FLAG, fludarabine, cytarabine, and granulocyte colony-stimulating factor; HCT, hematopoietic cell transplantation; HR, hazard ratio; MDS, myelodysplastic syndrome; MFC, multiparameter flow cytometry; MRD, measurable residual disease; NGS, next-generation sequencing; OS, overall survival; qPCR, real-time quantitative polymerase chain reaction; RIC, reduced-intensity conditioning. * MRD results were reported for responding patients. ^†^ HRs are shown with their 95% confidence intervals.
